# A high-risk Zika and dengue transmission hub: virus detections in mosquitoes at a Brazilian university campus

**DOI:** 10.1186/s13071-018-2883-8

**Published:** 2018-06-22

**Authors:** Alvaro E. Eiras, Simone F. Pires, Kyran M. Staunton, Kelly S. Paixão, Marcelo C. Resende, Hilcielly A. Silva, Isadora G. Rocha, Bruna A. Oliveira, Anderson M. Peres, Betânia P. Drumond, Scott A. Ritchie

**Affiliations:** 1 Department of Parasitology, Institute of Biological Sciences, Universidade Federal de Minas Gerais, Belo Horizonte, MG Brazil; 2 College of Public Health, Medical and Veterinary Sciences, James Cook University, Cairns, Australia; 3 Australian Institute of Tropical Health and Medicine, James Cook University, Cairns, Australia; 4 Department of Microbiology, Laboratório de Vírus, Institute of Biological Sciences, Universidade Federal de Minas Gerais, Belo Horizonte, Brazil

**Keywords:** *Aedes aegypti*, Dengue virus, Zika virus, GAT, Dissemination premises

## Abstract

**Background:**

Zika virus (ZIKV) and dengue virus (DENV) are mosquito-borne flaviviruses prevalent throughout tropical regions. Currently, management of ZIKV and DENV centers on control of the primary vector *Aedes aegypti.* This vector is highly anthropophilic and is therefore prevalent throughout densely urbanised landscapes. A new passive trap for gravid *Ae. aegypti* (Gravid Aedes Trap - GAT) was developed for mosquito surveillance. Here the different killing agents and the level of transmission of arboviruses that may occur in mosquitoes sampled by GATs are assessed for the first time.

**Methods:**

Gravid *Aedes* traps (GATs) were deployed at the Federal University of Minas Gerais campus, in Belo Horizonte, Brazil to sample *Ae. aegypti*. Three different killing agents were evaluated within the GATs: sticky cards, long-lasting insecticide-impregnated nets (LLINs) and canola oil. Traps were monitored weekly for 14 weeks then mosquito specimens were identified to the species level and *Ae. aegypti* catches were pooled and submitted to qRT-PCR assays for to DENV and ZIKV virus detection, followed by Bayesian phylogenetic analysis of the ZIKV. Additionally, comparisons of means were performed on transformed weekly catch data (*P* = 0.05, t-tests) with the *stats* package of the *R* statistical software.

**Results:**

In total, 1506 female *Ae. aegypti* were captured using GATs, with traps using sticky cards catching more mosquito than those using either LLINs or canola oil. Both ZIKV and DENV were detected in *Ae. aegypti* females captured over several weeks suggesting that this highly populated university campus may have served as a significant transmission hub. The infection rate for ZIKV was present in seven (8.5%) pools from four weeks while DENV was detected in four (4.9%) pools from four weeks. Phylogenetic analysis of ZIKV classified the strain as Asian genotype.

**Conclusions:**

The Federal University of Minas Gerais and similar organizations must strongly consider monitoring *Ae. aegypti* populations and reinforcing personal protection of staff and students during seasons of high mosquito activity.

## Background

Zika virus (ZIKV) and dengue virus (DENV) are responsible for grave health concerns throughout tropical regions [1, 2]. As platform technologies for the ZIKA vaccine are in development [3] and a vaccine for DENV 1-4 has only recently received approval for limited use in several countries [4], these arboviruses are currently still predominantly controlled *via* public health surveillance and control measures. *Aedes aegypti* is the primary vector for both ZIKV and DENV [5, 6], therefore surveillance measures often focus on detecting and monitoring populations of this highly anthropophilic mosquito [7, 8]. Of special concern to public health bodies are ignition premises which house large numbers of mobile people and dissemination premises which may facilitate the rapid dispersal of disease throughout the community [9]. While education facilities have been suggested to be dissemination premises for DENV [9], they are currently not considered to pose any greater risk of transmission for ZIKV than other institutions [10].

ZIKV, originally identified in Uganda in 1947, reappeared in 2007 in Yap and Micronesia, exploding throughout South America in 2016 and has recently emerged in India [11–13]. During the 2016 outbreak in South America ZIKV spread to 27 Brazilian states, including Minas Gerais [14]. While most ZIKV infections are subclinical, serious congenital malformations, such as microcephaly in newborns, have been associated with this disease [15, 16].

DENV, comprised of four serotypes (DENV 1-4), is the most significant arbovirus worldwide resulting in an estimated 390 million infections per year [17]. Being endemic in over 100 countries [2], this disease is ubiquitous throughout the tropics with a distribution linked to variances in rainfall, temperature and urbanization [17]. Disease manifestations associated with DENV vary greatly from asymptomatic/mild infections to severe shock syndrome with a case mortality rate of 1–2% [18].

As vaccines or antivirals for both ZIKV and DENV require further development [3, 4] control of these diseases relies on effective control of the primary vector *Ae. aegypti.* This highly anthropophilic mosquito is well-adapted to living in human environments [19]. It oviposits in artificial containers, harbours inside human dwellings and even preferentially feeds on humans [20]. Such behaviours result in *Ae. aegypti* being an extremely effective vector of human disease, especially in urbanised developing countries [21].

Effective vector population surveillance in urban areas is vital to monitor impacts from vector control strategies and to reveal potential transmission sources of new epidemics. A recent change in surveillance practices towards sampling adult *Aedes* occurred to better correlate vector presence with disease risk [22]. Responding to this need, the Gravid Aedes Trap (GAT) was recently developed to passively sample gravid *Aedes* mosquitoes [7]. These female mosquitoes are attracted to infusions set within black bucket bases as ovipositing sites [7].

Once the mosquitoes enter inside the GAT, various killing agents such as pyrethroid surface sprays are used to kill them [23]. While commonly used for vector control, insecticides can be hard to obtain and drive resistance within insect populations [22, 24]. There are however, a range of environmentally-friendly insecticide-free killing agents which have been developed to capture mosquitoes within the GAT, including sticky cards and canola oil [22, 25]. This study performed surveillance of an *Ae. aegypti* population within a Brazilian university utilising GATs with diverse killing agents to detect arboviruses transmitted by *Ae. aegypti* and compare the effectiveness of different killing agents.

## Methods

### Study area

The study was performed throughout the Institute of Biological Science (IBS) in the Federal University of Minas Gerais (UFMG) campus in the Pampulha District of Belo Horizonte, Brazil. Belo Horizonte is the sixth largest city in Brazil and UFMG accommodates approximately 56,000 students and staff.

### Trapping methodology

We deployed 40 GATs set on ground level outside within the IBS building complex. GATs were monitored weekly for 14 weeks (22 February - 19 May 2016), with no trapping on week nine. Traps using alternating killing agents were set in pairs > 20 m apart, with positions swapped each week to control for positional bias. At each point, two GATs were installed with approximately 2 m between them totalling 20 positions.

Three different killing agents were trialled within the GATs deployed: sticky cards, long-lasting insecticide-impregnated nets (LLINs) and canola oil. The sticky cards (14 cm long and 3.5 cm wide at the top margin and 7 cm wide at the bottom margin) were attached between the entry funnel and the inner wall of the translucent chamber to intercept mosquitoes flying between the funnel and trap wall (Silvandersson, Knäred, Sweeden). The LLINs (25 × 25 cm), treated with alphacypermethrin (4.8%), were supplied with the BG-GAT (Biogents AG, Regensburg, Germany). LLINs were placed loosely on the bottom mesh of the GAT head in a nested configuration. The final killing agent, canola oil (Purilev, Cargil Agricola S.A.) was applied as a thin coating inside the translucent chamber.

Traps were operated for a 14-week period, from February to May, and examined weekly. During weeks one to eight, GATs using sticky cards (sticky A) and LLINs were set, and from weeks 10–14 GATs with sticky cards (sticky B) and canola oil were deployed. Mosquito specimens collected were identified to species and *Ae. aegypti* catches were pooled (*n =* 20/pool) then virus detection was performed with DENV and ZIKV qRT-PCR assays.

### RNA extraction

The collected mosquitoes were stored in 250 μl of guanidine solution and kept at room temperature until RNA extraction [26]. All *Ae. aegypti* mosquitoes collected were grouped in 82 different pools and were tested using the DENV and ZIKV qRT-PCR assay. The pools, containing up to 20 mosquitoes, were macerated manually using a sterilized pestle then centrifuged for 10 min at 10,000× *g* at room temperature and 140 μl of the supernatant were used to RNA extraction with the QiaAmp Viral RNA Extraction Kit (Qiagen, Hilden, Germany), according to the manufacturer’s protocol. RNA was stored at -80 °C until use. Viral culture supernatant of DENV 1-4 and ZIKV were used for RNA extraction and as positive controls of reactions.

### Molecular investigation of ZIKV and DENV

RNA was amplified using qRT-PCR assay with a StepOne Real-Time PCR System (Applied Biosystems, Foster City, CA, USA) and SuperScript™ III Platinum™ One-Step qRT-PCR Kit (Invitrogen, Carlsbad, California, USA) for DENV detection and ZIKV was amplified using Power SYBR green RNA-to-Ct (Applied Biosystems, California, USA). For both viral reactions, virus RNA was included as an external control in every qRT-PCR run. Primers and probes used for DENV all, DENV 1-4 and ZIKV virus detection was described by [27–29] respectively. DENV all and serotypes DENV 1-4 reactions were carried out in 25 μl reaction mixtures including 4 μl of nucleic acid sample, 12.5 μl of 2× Premix, 0.5 μl of SuperScript III Platinum Taq mix. The thermocycling parameters were: a step for 30 min at 50 °C, followed by 95 °C for 2 min and 40 cycles at 95 °C for 15 s and 60 °C for 1 min. Samples generating a threshold cycle (Ct) of > 37 in duplicates were considered negative. The primers and probes concentrations used by each case were: DENV all (160 nM forward and reverse primers, 80 nM TaqMan probe), DENV-1 (200 nM forward and reverse primers, 100 nM TaqMan probe), DENV-2 and DENV-4 (150 nM forward and reverse primers, 75 nM TaqMan probe) and DENV-3 (250 nM forward and reverse primers, 120 nM TaqMan probe).

All ZIKV reactions were performed by adding 4 μl of RNA template in 10 μl of reaction mix including 5 μl of Power SYBR green RNA-to-Ct (Applied Biosystems, Foster City, California, USA), 300 nM of each primer and 0.12 μl of AmpliTaq. The reaction conditions consisted of a 30 min at 48 °C and then 10 min at 95 °C, followed by 40 cycles at 95 °C for 15 s and 60 °C for 3 s. Samples generating a melt curve with Temperature Melting near 81 °C were considered positive. NS5 and 3'NC regions of positive mosquito pools for ZIKV and DENV, respectively, were amplified and sequenced using specific primers. The amplicons were purified (PureLink™ Quick Gel Extraction and PCR Purification Combo Kit da Invitrogen) and sequenced in both directions by dideoxi method, using specific primers. The sample sequencing was performed by Myleus Biotechnology Ltda using capillary electrophoresis on ABI 3730 instrument, using BigDye v3.1 and POP7 polymer. Raw sequence quality was assessed using PHRED and contigs were generated using CAP3 implemented in a platform for electropherogram quality analysis (http://asparagin.cenargen.embrapa.br/phph/). Sequences from primers were removed from final contigs and the final amplicons were compared to sequences deposited in GenBank. Given their small sizes, dengue sequences (65 bp) were not used for phylogenetic inferences. Sequence alignments were performed using the MultiAlin by Florence Corpet [30].

### Phylogenetic analyses

Sequences of ZIKV were also used for phylogenetic inferences based on maximum likelihood or Bayesian methods. Bayesian phylogenetic analyses of ZIKV were performed and ZIKV sequences from African and Asian genotypes were retrieved from GenBank [31]. Nucleotide sequences (*n* = 90) were aligned using MAFFT [32]. Phylogenetic trees, based on maximum likelihood or Bayesian methods, were reconstructed. Maximum Likelihood trees were reconstructed using PhyML [33]. The nucleotide substitution model TN+G was selected using SMS [34] and for tree search, the SPR branch-swapping algorithm was used followed by the approximate likelihood-ratio test (aLRT) to assess the support of branches. Bayesian analysis was carried out using the BEAST package v.1.8.2 [34] with Markov Chain Monte Carlo algorithms. Input files for BEAST were created with BEAUTi 1.8.2. One hundred million chains were run and the trees were sampled at each of 10,000 steps and then summarized in a maximum clade credibility tree using TreeAnotator v.1.8.2 [35]. The final trees were visualized in FigTree v.1.4.3 [36].

### Statistical analysis

Comparisons of means were performed on transformed weekly catch data [log(x+1)] of female *Ae. aegypti* using independent sample two-tailed t-tests (*P* = 0.05) with the *stats* package available in the *R* statistical software (ver 3.3.3.). Minimum infection rates for ZIKV and DENV were calculated using the pooled infection rate program (PooledInfRate, version 4, Center for Disease Control and Prevention, Fort Collins, CO [37]).

## Results and discussion

**Fig. 1:**
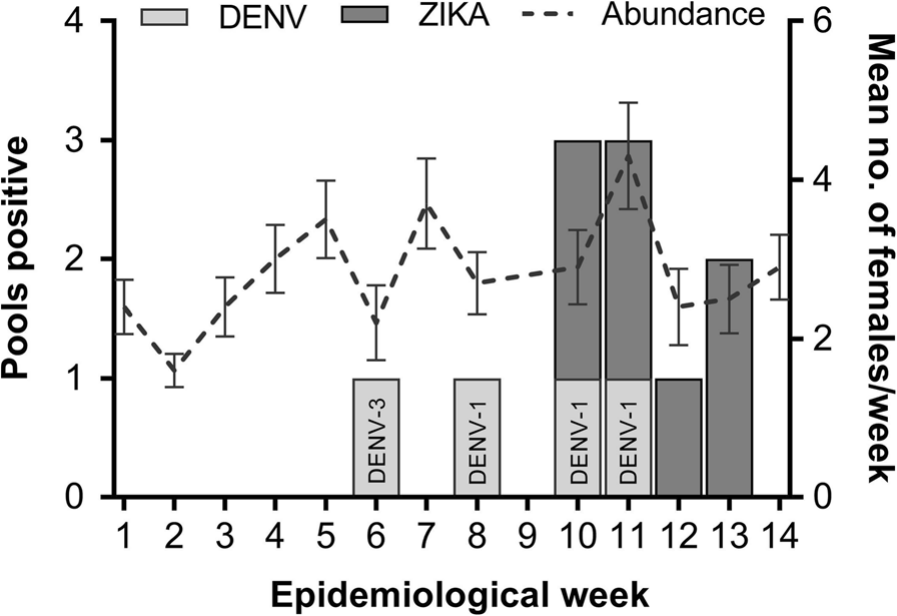
Detection of DENV (serotype within bar) and ZIKV with trap catches (mean number per week ± SE) of female *Ae. aegypti* across sampling effort. *Note*: Data were not collected for week nine

A total of 1564 mosquitoes were captured in the GATs. Among these, 58 were identified as *Culex quinquefasciatus* and 1506 as *Ae. aegypti*. The minimum infection rate for ZIKV was 4.99‰ as it was present in seven (8.5%) pools from four weeks (Fig. 1). DENV displayed a minimum infection rate of 2.80 ‰ and was detected in four (4.9%) pools from four weeks.

**Fig. 2:**
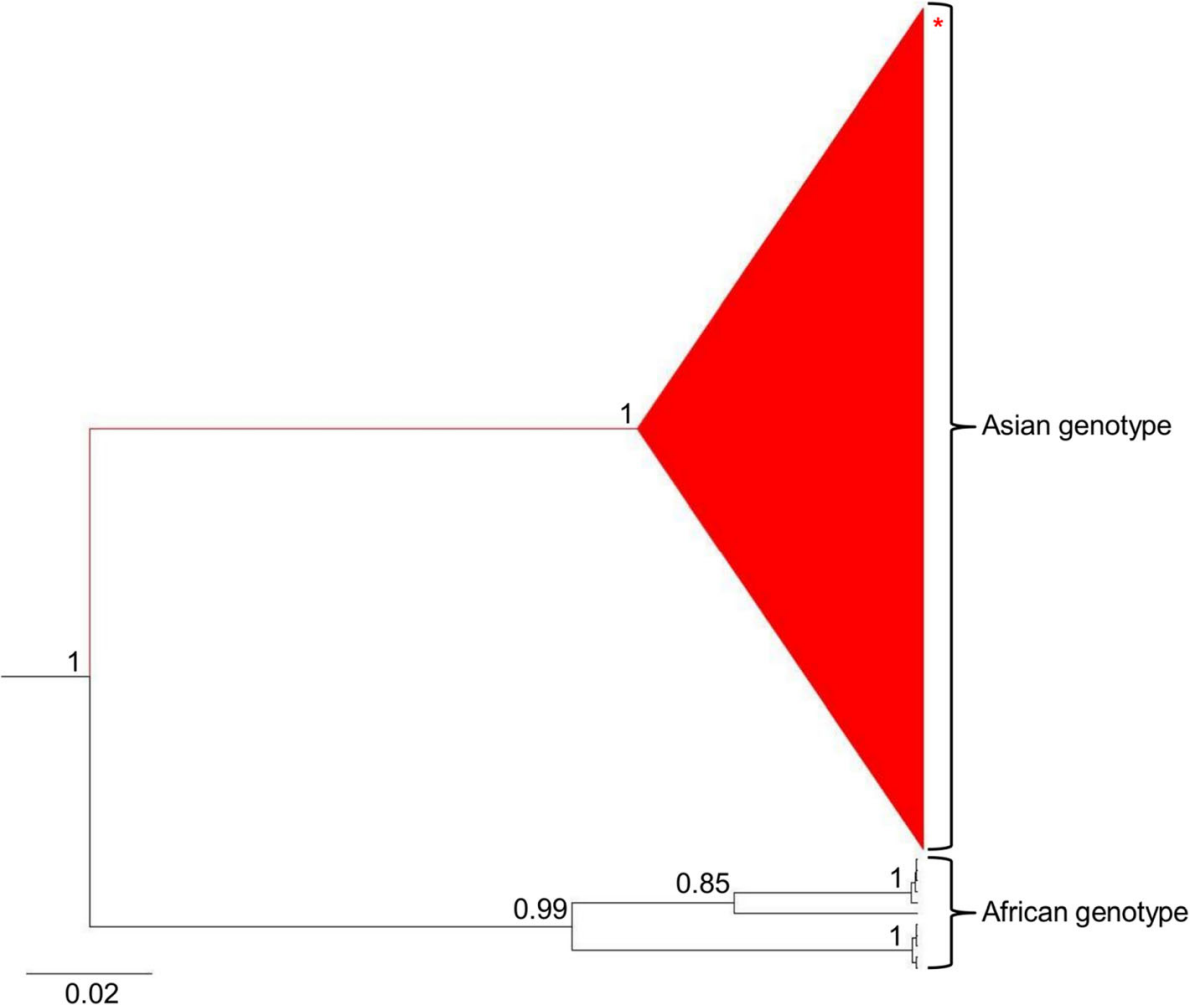
Bayesian phylogenetic analysis of ZIKV. The evolutionary history was inferred by using the Tamura-Nei model plus gamma distribution. The tree is drawn to scale, with branch lengths measured in the number of substitutions per site. The analysis involved 90 nucleotide sequences with a total of 148 positions in the final dataset. Values of posterior probability are shown at the nodes. The clade containing strains from an Asian genotype is indicated in red. Strains from Asia, Europe and America from 2012 up to 2016 were collapsed for clarity. The red asterisk indicates the relative position of six ZIKV strains detected in pools of mosquito in this study. Evolutionary analyses were conducted with BEAST package v.1.8.2

For two weeks, both ZIKV and DENV were simultaneously detected on campus. Indeed, multiple serotypes of dengue were detected-DENV-3 at epidemiological week 6 and DENV-1 at weeks 8, 10 and 11 (Fig. 1). Positive samples were sequenced and confirmed the presence of ZIKV (in seven samples) and DENV (DENV-1 in four samples) on campus. The amplicons presented the highest similarity values when compared to ZIKV NS5 (97–99%) and DENV-1 3'NC (95–100%) sequences. ZIKV sequences obtained from six pools were identical to each other and comprised of 148 nt (position 1489 to 1636 of the NS5 gene). After the analyses of maximum likelihood trees (data not shown) and Bayesian trees (Fig. 2) all the strains studied here grouped within strains from Asian genotype. These data confirmed the detection of ZIKV from Asian genotype in mosquitoes from the campus. Given the small size of nucleotide sequences, we were not able to further investigate the origin or the evolution patterns of these viral strains.

**Fig. 3:**
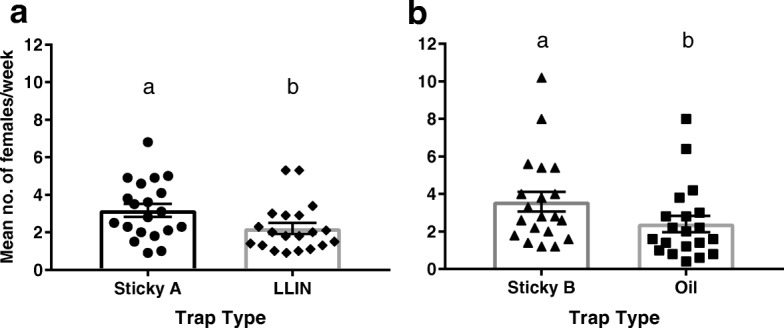
Mean number of female *Ae. aegypti* caught per week (mean ± SE) from different trap types. **a** Comparison of GATs with sticky cards and LLIN killing agents. **b** Comparison of GATs with sticky cards and oil killing agents. Labels indicate significant groupings (*P* < 0.05, t-test, *n* = 20)

GATs using sticky cards as a killing agent were found to sample more *Ae. aegypti* than traps using LLIN (*t* = 2.24, *df* = 38, *P* = 0.031, *n* = 20) or canola oil (*t* = 2.11, *df* = 38, *P* = 0.042, *n* = 20; Fig. 3).

To our knowledge, this is the first record of ZIKV in *Ae. aegypti* in the State of Minas Gerais, Brazil. From monitoring the campus for only 13 weeks, a combined minimum infection rate of 8.04‰ was detected, with 13.4% of pools positive for either ZIKV or DENV. The minimum infection rate detected for ZIKV was comparable to that detected among *Ae. aegypti* in Senegal where the disease was suggested to be maintained in vertebrate reservoirs [38]. The DENV minimum infection rate was consistent with several concurrent cases of DENV infection occurring in the UFMG population and a recorded incidence rate of 6521 per 100,000 in Belo Horizonte [39]. In 2016, the incidence rate of ZIKV in Belo Horizonte was 37.82 per 100,000. As both ZIKV and DENV display such high rates of subclinical infection [40, 41] monitoring vector infection rates is a critical control measure.

These results indicate that this highly populated university campus may have served as a significant transmission hub for DENV and ZIKV in 2016. Indeed, 542 ZIKV cases were confirmed in 2016, including 59 within Belo Horizonte’s Pampulha District where UFMG is located [39].

University populations may be especially vulnerable to complications from ZIKV, which has even been suggested to be transmitted sexually [42, 43] as high proportions of young adults attend these facilities. While educational institutions have long been considered to be dissemination premises for DENV [9], they are stated to be unlikely to have higher risks of transmission for ZIKV than other facilities [10]. However, the high detection rates of both ZIKV and DENV in these buildings at university campus, combined with the large population of students who are likely to be very mobile, indicates that this organization may indeed be an effective dissemination premise.

Current control measures for both DENV and ZIKV employed within the UFMG include 250 GATs throughout the campus for adult mosquito surveillance, monitoring sump pits in all buildings and communication to students and staff *via* the university media.

This study indicated that GATs using the insecticide-free sticky cards as killing agents caught more *Ae. aegypti* than those using either LLINs or canola oil. These results differ to a previous study, which did not find differences in catch rates between GATs using these killing agents [22]. However, our findings are consistent with the study by Heringer et al. [22] in suggesting that the insecticide-free sticky cards are suitable replacements for traditional insecticides. The use of sticky cards coupled with strategies for rapid and specific viral detection would certainly improve arboviruses surveillance and control programmes. Two recent studies has shown that NS1 rapid tests was able to detect DENV in experimentally and naturally infected mosquitoes [44, 45]. While it has great potential, especially for rapid public health virus surveillance of mosquitoes, the method needs to be evaluated in mosquito and viral surveillance programmes sampling wild mosquito populations. Furthermore, efforts should be made to obtain DENV RNA from some mosquito pools for sequencing and phylogenetic study, as well as to determine the viral serotype. The efficacy of sticky cards in this study, combined with their environmentally friendly qualities and the fact that they do not drive insecticidal resistance, suggests that they are very attractive tools for future deployments of GATs as vector surveillance traps.

## Conclusions

Educational institutions, such as the UFMG, may benefit from implementing effective vector surveillance programs as well as reinforcing personal protection of attendees when mosquitoes are most active. Additional preventative control measures should also be reviewed including screening doors and windows and covering key containers such as sump pits to reduce mosquito production from larval habitats [36].
